# Addressing the Language Binding Problem With Dynamic Functional Connectivity During Meaningful Spoken Language Comprehension

**DOI:** 10.3389/fpsyg.2018.01960

**Published:** 2018-10-12

**Authors:** Erin J. White, Candace Nayman, Benjamin T. Dunkley, Anne E. Keller, Taufik A. Valiante, Elizabeth W. Pang

**Affiliations:** ^1^Neurosciences and Mental Health, Sick Kids Research Institute, Peter Gilgan Centre for Research and Learning, The Hospital for Sick Children, Toronto, ON, Canada; ^2^Epilespy Research Program of the Ontario Brain Institute, Toronto, ON, Canada; ^3^Department of Diagnostic Imaging, The Hospital for Sick Children, Toronto, ON, Canada; ^4^Department of Medical Imaging, University of Toronto, Toronto, ON, Canada; ^5^Krembil Research Institute, University Health Network and Toronto Western Hospital, Toronto, ON, Canada; ^6^Division of Neurosurgery, Department of Surgery, University of Toronto, Toronto, ON, Canada; ^7^Division of Neurology, The Hospital for Sick Children, Toronto, ON, Canada

**Keywords:** speech comprehension, dynamic functional connectivity, phase synchrony, PLI (phase lag index), gamma, theta

## Abstract

During speech, how does the brain integrate information processed on different timescales and in separate brain areas so we can understand what is said? This is the language binding problem. Dynamic functional connectivity (brief periods of synchronization in the phase of EEG oscillations) may provide some answers. Here we investigate time and frequency characteristics of oscillatory power and phase synchrony (dynamic functional connectivity) during speech comprehension. Twenty adults listened to meaningful English sentences and non-sensical “Jabberwocky” sentences in which pseudo-words replaced all content words, while EEG was recorded. Results showed greater oscillatory power and global connectivity strength (mean phase lag index) in the gamma frequency range (30–80 Hz) for English compared to Jabberwocky. Increased power and connectivity relative to baseline was also seen in the theta frequency range (4–7 Hz), but was similar for English and Jabberwocky. High-frequency gamma oscillations may reflect a mechanism by which the brain transfers and integrates linguistic information so we can extract meaning and understand what is said. Slower frequency theta oscillations may support domain-general processing of the rhythmic features of speech. Our findings suggest that constructing a meaningful representation of speech involves dynamic interactions among distributed brain regions that communicate through frequency-specific functional networks.

## Dynamic Functional Connectivity During Meaningful Spoken Language Comprehension: Addressing the Language Binding Problem

How it that we can create a coherent and meaningful representation of a multi-word utterance when different features of the speech signal are processed by separate brain areas and at different timescales as the speech signal unfolds? is This so-called “language binding problem” continues to be a central question in the neuroscience of language (Hagoort, 2005). Functional connectivity, mediated by the phase synchronization of neuronal oscillations, provides a window into the brain’s language networks (Weiss and Mueller, 2003; Giraud and Poeppel, 2012) and may provide a mechanism to help address the language binding problem. However, relatively few studies have investigated functional connectivity during speech perception (e.g., Luo and Poeppel, 2007; Kikuchi et al., 2011). The goal of this study is to better understand the time and frequency characteristics of the functional networks that support meaningful spoken language processing in the brain.

Many previous studies have used event-related potentials (ERPs) to examine the neural basis of speech comprehension. The high temporal precision of ERPs has been crucial for investigating how language processing unfolds in the brain over time. ERPs, however, measure localized brain responses and cannot reveal the dynamic interactions between brain areas that support language comprehension in real-time. With time-frequency analysis of EEG oscillations, one can measure both changes in local brain activity and long-range communication among distributed brain regions during language processing. Oscillatory power (amount of energy at a particular frequency) is thought to reflect local neuronal activity, which may be due to the number (or strength) of neurons firing at a particular frequency, as well as how synchronous their firing is (Cohen, 2014). Additionally, a correlation in the phase of oscillations at two different electrodes (i.e., coordinated fluctuations of rhythmic excitability of neural populations recorded from different electrodes) is thought to reflect long-distance synchronization, and thus interaction, among distributed brain regions even if those regions are not physically connected (Buzsáki and Wang, 2012; Siegel et al., 2012; Fries, 2015). The brain’s ability to change the extent to which neurons in different areas synchronize their patterns of firing is thought to be a mechanism by which it coordinates and integrates the flow of information within a network of participating structures (Bastos and Schoffelen, 2016). Dynamic functional connectivity, as measured through changes in cross-trial phase synchronization over time, has been used to investigate the brain networks supporting many aspects of sensory and cognitive processing (Rodriguez et al., 1999; Singer, 2007; Siegel et al., 2012; Fries, 2015). As of yet, however, it has been underused to examine the brain networks supporting speech perception.

Here we explore the time and frequency characteristics of both oscillatory power and phase synchrony (dynamic functional connectivity) during meaningful spoken sentence processing. Specifically, we ask whether there is a difference in the overall phase synchronization of EEG oscillations when healthy native English speaking adults listen to meaningful English sentences compared to non-sensical “Jabberwocky” sentences, which lack semantic content. In Jabberwocky sentences, English open class words (nouns, verbs, adjectives, adverbs) are replaced with pseudo-words that, while obeying English phonotactic rules, are void of meaning (Carroll, 1883; Yamada and Neville, 2007). Without meaningful lexical-semantic content, both the memory retrieval and the binding stages of language comprehension that unify semantic with syntactic, and phonological information are disrupted (Hagoort, 2005). Jabberwocky uses English closed-class words (e.g., articles, prepositions) however, which is thought to allow English listeners to create a rudimentary structural representation of the sentence and engage in syntactic processing, even in the absence of meaningful semantic information (although see Hahne and Jescheniak, 2001 and Yamada and Neville, 2007 for alternative views as to whether syntactic processing recruits identical neurocognitive processes without semantic information). Comparing English to Jabberwocky thus allows us to investigate the brain processes specific to meaningful speech comprehension and integration, while controlling for other levels of language processing (e.g., phonology, syntax). We predict that semantic integration will be reduced or absent while listening to Jabberwocky compared to English sentences and this will be indexed by a reduction in overall oscillatory phase synchrony.

Phase synchronization of EEG oscillations can occur at different frequencies. These frequencies reflect the rate at which neurons alternate between a state in which they are more or less excitable, likely to fire and efficient at processing incoming information (Schroeder et al., 2008). The results of previous studies suggest that oscillations in the gamma (30–80 Hz) and theta frequency range (4–7 Hz) may be important in speech processing. For example, in terms of local power changes, greater power was seen in the middle gamma frequency range (defined as 55–75 Hz) when participants listened to their native language compared to a foreign language or speech played backward, whereas listening to both languages resulted in a power increase in the theta frequency range (4–7 Hz; Peña and Melloni, 2012). Increased phase synchronization in the theta frequency range was also reported when participants listened to normal speech compared to speech that was degraded to the point where it was unintelligible (Luo and Poeppel, 2007). Moreover, Molinaro et al. (2013) reported increased phase synchronization in both theta and gamma frequency bands when participants read words presented in highly constraining lexical/semantic contexts that pre-activated the expected words’ lexical/semantic representations compared to words in less constraining contexts that did not benefit from such anticipatory semantic preparation. By investigating both local and long-range oscillatory responses (power and phase synchrony, respectively), the present study extends these findings to better elucidate the brain networks supporting the comprehension and integration of meaning in speech. Based on previous findings, we expected to see increased oscillatory power and phase synchrony (functional connectivity) in gamma and theta frequency ranges when participants listened to English compared to Jabberwocky speech.

## Materials and Methods

### Participants

Twenty right-handed, university-educated native English speakers (21–36 years; 11 females) participated. All reported normal vision, hearing and neurological health and provided informed consent. This study was approved by the Research Ethics Board at the Hospital for Sick Children.

#### Materials and Procedure

EEG was recorded in a quiet room while participants listened to naturally spoken sentences via headphones set to a comfortable volume level. Here, two sentence conditions were analyzed: regular English sentences (e.g., “They jump off their beds and onto the floor”) and non-sensical Jabberwocky sentences in which pseudo-words replaced all open-class (content) words (e.g., “Klee sma nim falc chure in her molall”). Pseudo-words were created by substituting phonemes of words from correct English sentences with a different phoneme (vowels were replaced by another vowel, consonants by another consonant with the same manner of articulation as long as this yielded permissible English consonant clusters). The initial phonemes of open-class words were retained, as were all closed-class words. Sentences were 5–15 words in length (mean sentence length was 3.26 ± 0.82 s, ranging from 2.113 to 6.133 s, 85% of the sentences were greater than 2.5 s). The interval between sentences ranged from 1.5 to 2.5 s. The process for creating Jabberwocky sentences is described in detail in Yamada and Neville (2007). In total, participants heard 50 of each sentence type, which were pseudo-randomly presented with other English sentences that were correct or contained semantic, morphosyntactic, or phase structure violations (results described elsewhere).

The findings of our study are intended to inform future investigation with children, for whom engaging experimental paradigms are especially important. As such, all sentences were embedded in ongoing narratives and accompanied by 5 engaging animated movies, each approximately 5 min long. Each movie told a story of Pingu the penguin, with the experimental sentences (10 of each condition per film), describing scenes in the movie. One narrator told each story (3 female, 2 male). Before beginning the experiment, participants were told that while some of the narrator’s sentences might sound strange, they shouldn’t think too much about them and instead try to enjoy the movies. No response was required. Most participants reported finding the movies and overall experiment to be entertaining, that the simultaneous presentation of the movies and sentences did not deter from their comprehension of the individual English sentences, and that the Jabberwocky sentences were indeed meaningless. The simultaneous presentation of movies with the experimental sentences highlighted the main contrast of this experiment: the processing of meaningful versus meaningless speech. For the English sentences, lexical-semantic access was facilitated both by what the participants heard and saw in the corresponding animations (e.g., “Pingu hit his fork on the table” was accompanied by the same scene). Conversely, for the Jabberwocky sentences, lexical-semantic access was interrupted, both by the lack of meaning in the sentences participants heard and the mismatch this formed with what they saw in the animations (e.g., “Klee sma nim falc chure in her molall” was accompanied by an animation of Pingu rolling a ball).

### EEG Recording and Processing

Continuous EEG data were recorded from 64 cap-mounted electrodes (1000 Hz sampling, 0.01–200 Hz filter, referenced to an electrode between Cz and CPz for acquisition, impedance < 10 kΩ) using a NeuroScan v4.5 Synamps2 amplifier (Compumedics, El Paso, TX, United States). Vertical and horizontal eye movements were monitored.

Data processing was done using the Fieldtrip toolbox in Matlab (Oostenveld et al., 2011). Data were low-pass filtered at 100 Hz, re-referenced to the average of all EEG channels, epoched into individual trials relative to sentence onsets, de-trended to remove slow-shifts and then downsampled to 500 Hz. Artifact rejection included rejection of trials with absolute amplitude greater than 200 μV, as well as independent component analysis (ICA; Jung et al., 2000) to remove eye movements (e.g., due to blinks and movements related to watching the movies) and heart artifacts. This resulted in the removal of relatively few trials (mean 3.5 trials across participants and sentence conditions), with no difference between conditions (*p* > 0.10).

### EEG Analysis

Trial-by-trial data were transformed by z-scores, filtered into canonical frequency bands (theta 4–7 Hz, alpha 8–14 Hz, beta 14–24 Hz, and gamma 30–80 Hz; single pass FIR filter created using a hamming window). Phase estimates were obtained using the Hilbert transform from ±3 s around sentence onsets. Functional connectivity was measured using Phase Lag Index calculated across trials (PLI; Stam et al., 2007) and was programmed in Matlab (Mathworks, Inc). PLI measures the cross-trial phase synchrony between oscillations at two electrodes with a temporal lag, thereby avoiding spurious effects of volume conduction (i.e., activity from one underlying neural generator is recorded at two electrodes and mistaken for synchrony; Cohen, 2014). The PLI is most sensitive to -/+90°lags/leads or 1/2π rad, thus by attenuating zero-phase correlations, the PLI is more robust to spurious correlations driven by common sources (e.g., due to volume conduction) compared to other measures of phase synchrony (e.g., coherence, phase locking values) and is therefore a preferred method for interrogating EEG connectivity. This resulted in electrode x electrode connectivity matrices for each time point, frequency band, and sentence condition. A time series of global connectivity was computed by averaging PLI values (strength) across electrodes (Doesburg et al., 2016; Mennella et al., 2017).

Differences in connectivity while listening to English and Jabberwocky were evaluated in three steps. First, a paired two-tailed *t*-test compared global connectivity values for English and Jabberwocky at each time point. This was done for each frequency band separately. Then, to control for multiple comparisons and set an objective statistical threshold for determining how many consecutive time points must show a significant condition difference (*p* < 0.05) to be considered meaningful, a cluster-based permutation test (1000 permutations shuffled across conditions, *p* < 0.05) was run for each frequency band between -0.5 and 2.5 s (Cohen, 2014). This gave a distribution of cluster lengths (i.e., stretches of time points for which a difference between English and Jabberwocky could occur by chance), as expected under the null hypothesis. The 97.5th percentile of this distribution was set as the threshold value against which we compared true condition differences in connectivity. Contiguous stretches of significant differences that were longer than the threshold were considered to be time windows when global connectivity was significantly different for English and Jabberwocky. This conservative approach reveals robust differences prolonged in time which span canonical frequency ranges. Finally, to explore which electrode-electrode connections contributed most to the global connectivity effect, the connectivity strength of each electrode pair was averaged within the time window, a difference between conditions was calculated, and the top 1 and 5% of electrode pairs were plotted on a topographical map. Thus, for each frequency band, “global connectivity” shows *when* and at which *frequency* there is a prolonged difference in connectivity (phase synchrony) between correct and violation sentences and “electrode-electrode connectivity” shows *where* (between which electrodes) this difference is the strongest. In the absence of expected between-condition effects, a *post hoc* analysis was conducted to explore significant connectivity increases as a function of sentence listening. For this we compared active (500 ms–1 s) and baseline (-l s to -500 ms) windows for both English and Jabberwocky sentences.

Oscillatory power was computed using Morlet wavelets (5 cycle width, 3 SD Gaussian time window function) on single trials between 1–80 Hz in 1 Hz and 50 ms steps, ±3 s surrounding critical word onsets using Fieldtrip software (Oostenveld et al., 2011). English and Jabberwocky trial data were averaged separately, and expressed as an increase or decrease relative to the decibel power within a -500 to -200 ms baseline (Cohen, 2014). Statistical analyses were performed using cluster based permutation tests to control for multiple comparisons (Maris and Oostenveld, 2007). English and Jabberwocky data were each averaged over frequencies and into the canonical bands (theta, alpha, beta and gamma) and were compared using two-tailed paired *t*-tests conducted at each electrode and time point between 0 and 2.5 s. Comparisons that exceeded a significance level of 0.05 were grouped into clusters, their t-statistics were summed and compared to a null distribution (created by 1000 random data partitions). Any cluster-level test statistic that fell into the highest or lowest 2.5th percentile was considered significant.

## Results

### Functional Connectivity

**FIGURE 1:**
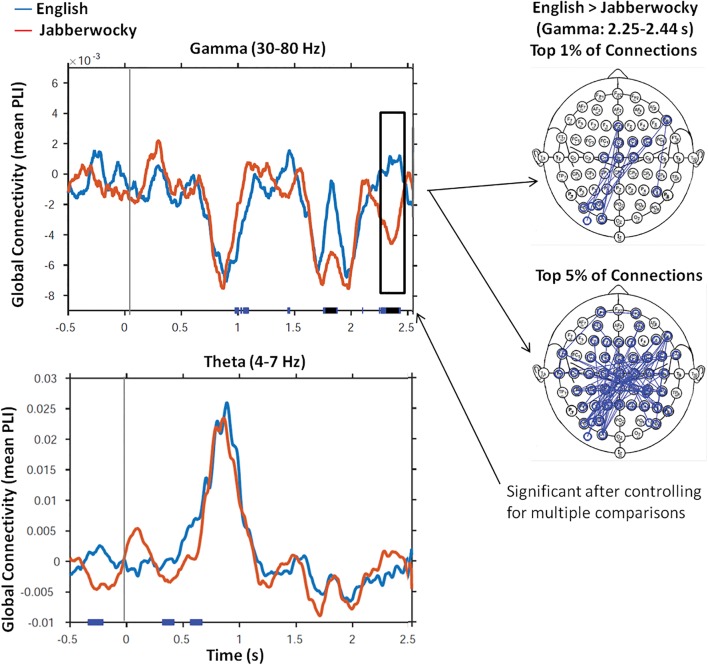
Global Connectivity. This figure shows time series of global connectivity (mean PLI values) for English (blue) and Jabberwocky (red) sentences for the gamma and theta frequency bands. Results of the running *t*-tests that compared mean PLI values for English versus Jabberwocky at each time point are presented along the x-axis of each figure (blue: *p* < 0.05; black: *p* < 0.01). Only the effect in the gamma frequency range (30–80 Hz) remained significant after controlling for multiple comparisons (*p*_corr_ < 0.05). The head maps show the electrode – electrode connections that contributed most to the gamma connectivity effect in the 2.25–2.44 s time window.

**Figure 1** shows the time series of global connectivity (mean PLI values) from the onset of both English and Jabberwocky sentences for the gamma and theta frequency bands. The plots show two striking effects: a between condition effect in gamma, and an increase from baseline effect in theta. First, there was a significant difference (*p*_corr_ < 0.05) between conditions in the gamma frequency range (30–80 Hz). In gamma, significantly greater connectivity was seen for English over Jabberwocky 2.25–2.44 s after sentence onset. This effect was driven mostly by connections among left posterior and vertex regions. The second notable effect was a large increase in global connectivity in theta (4–7 Hz) around 0.5–1 s after both English and Jaberwocky sentence onsets (note difference in scale for theta), with no significant differences between conditions. For both English and Jabberwocky sentences, comparisons of active window (500 ms to 1 s) versus baseline (-l to -500 ms), revealed significant increases in theta connectivity for both sentence types (*p*_corr_ < 0.05).

### Oscillatory Power

**FIGURE 2:**
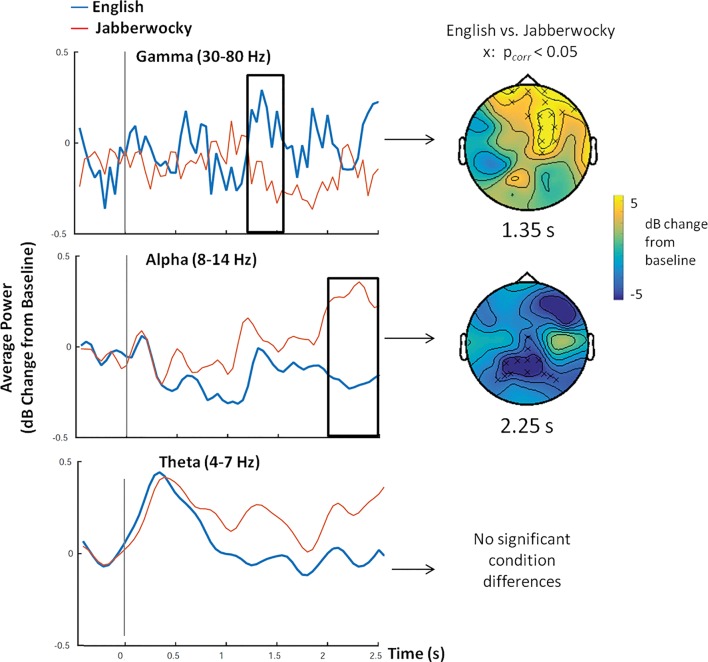
Oscillatory Power. Time series of oscillatory power for gamma (30–80 Hz), alpha (8–13 Hz) and theta (4–7 Hz) frequency bands over time, averaged over all electrodes for English (blue) and Jabberwocky (red) sentences. The head maps show topographical results (at a time point that was determined to be representative of the whole window based on visual inspection) of the cluster-based permutation test where a significant difference (x denotes *p_corr_* < 0.05) between conditions was observed for gamma and alpha frequencies only. In gamma, significantly more connectivity was seen for English sentences between 1.25 and 1.55 s that was largest at frontal electrodes. In alpha, significantly less connectivity was seen for English sentences between 2 and 2.65 s that was largest at frontal and posterior electrodes. In theta, a large increase in oscillatory power was seen for both English and Jabberwocky directly after sentence onset (0 ms), with no significant condition differences (*p* > 0.10).

**Figure 2** shows average power within gamma, alpha and theta frequency bands over time for English and Jabberwocky sentences, as well as results of the permutation test that revealed clusters of significant condition differences These plots show three notable effects: greater gamma band power and less alpha band power for English compared to Jabberwocky sentences, and increased theta band power around sentence onset that was similar for both conditions. These effects were confirmed by permutation tests run for each frequency band over 0–2.5 s. For the gamma frequency band, this revealed a significant positive cluster 1.25 –1.55 s after sentence onset (max sum = 145.50, *p* < 0.05) that was most prominent at frontal and midline electrodes. For the alpha frequency band, this test revealed a marginally significant negative cluster 2–2.5 s after sentence onset (max sum = -288.07, *p* < 0.08). Follow-up analyses conducted over a narrower 2–3 s time window revealed significantly less alpha band power for English between 2 and 2.65 s (max sum = -291.57, *p* < 0.03; **Figure 2**). This negative cluster was most prominent at midline central electrodes. No significant differences between English and Jabberwocky were found for theta or beta frequency ranges (*p*> 0.10). However, as can be seen in **Figure 2** both English and Jabberwocky showed an increase in theta band power around sentence onsets. For both English and Jabberwocky sentences, comparisons of active window (0–500 ms) versus baseline (-l s to -500 ms), revealed significant increases in theta power for both sentence types (English: *p* < 0.05; Jabberwocky: *p* < 0.001).

## Discussion

The main finding from this study was greater functional connectivity (phase synchrony) and oscillatory power in the gamma frequency range (30–80 Hz) when participants listened to meaningful English sentences compared to non-sensical Jabberwocky sentences. An increase in theta power and phase synchrony was also observed, but was similar for both English and Jabberwocky. These findings correspond to the power results of Peña and Melloni (2012), who also found greater gamma band power when participants listened to their native language compared to a foreign language with a similar time signature as found here (around 1 s after sentence onset). Additionally, these authors report increased theta band power directly following sentence onset for both native and foreign languages, similar to our findings for English and Jabberwocky. Our results extend these findings to global functional connectivity (phase synchrony) as well, to show that not only does the processing of meaningful speech modulate local neuronal activity, but it also changes the coordination of frequency-specific activity from distributed neuronal populations. Together, these finding suggest that oscillations in the gamma frequency range in particular may reflect a neuronal mechanism for integrating meaning during speech processing and a functional network underlying language comprehension.

More broadly, our results add to the growing literature showing a relationship between synchronous oscillations in the gamma frequency range and a variety of sensory and cognitive integrative functions, including perceptual grouping, maintaining information in short term memory and multi-sensory integration (Singer, 2007). Brief periods of synchronization in the gamma frequency range appear to act as an integrative mechanism that brings together the activity of widely distributed neuronal assemblies into a coherent network to support cognitive and perceptual processing (Rodriguez et al., 1999). Termed the “binding by synchrony” hypothesis, the idea is that two brain regions that consistently oscillate in synchrony are communicating with each other within a network, even if those areas are not physically connected (Siegel et al., 2012; Fries, 2015). High frequency oscillations in the gamma range (30–80 Hz and faster) appear to be most tightly linked with such network communication because a cycle of gamma corresponds to the time course of excitatory post-synaptic events (Jensen and Mazaheri, 2010). Our findings point to an additional role for gamma band phase synchronization (functional connectivity) and power in the retrieval and integration of meaning in speech.

Interestingly, the timing and distribution of local power and global phase synchrony effects in the gamma frequency band were different: whereas power effects were seen 1.25–1.55 s after sentence onset over prominently frontal electrodes, phase synchrony effects occurred later, between 2.25 and 2.44 s, and were largely due to interactions among left posterior and vertex regions. One might have predicted that frequency-specific network communication would occur at the same time or even precede local power effects in the corresponding frequency bands. However, the findings of the current study, as well as others, suggest no simple relation exists between local power and long-range phase synchronization effects (Donner and Siegel, 2011; Musall et al., 2012). For example, Hipp et al. (2011) report a dissociation between local power and phase synchrony in terms of timing, presence, and distribution of frequency-specific effects during an audiovisual perception task, and only phase synchronization predicted participants’ perceptions during the task. It may be that distant cortical sites synchronize their activity in a frequency-specific way, without necessarily corresponding to changes in local neuronal activity, and vice versa. In the present study, it could also be that a local increase in the number of neurons firing synchronously at the gamma frequency band for English (manifested by increased power) later contributed to greater global phase synchronization. In any case, it highlights the need for future work, examining event-related changes to both oscillatory power and phase synchrony, to better understand this relation.

At the same time and with a similar topographical distribution as the increased gamma phase synchronization, we observed *reduced* oscillatory power in the alpha frequency range (8–13 Hz) for English sentences. Alpha oscillations have been linked to attention and executive functioning and are thought to support both the inhibition of task-irrelevant and activation of task-relevant processing (Palva and Palva, 2011). Additionally, pulses of alpha activity may regulate cognitive and sensory processing through their inverse relationship with gamma oscillations, as proposed in the “gating by inhibition” hypothesis (Jensen and Mazaheri, 2010). By this account, decreased alpha activity allows excitatory neurons oscillating in the gamma frequency range to synchronize their firing patterns across task-relevant brain areas, whereas increased alpha activity temporarily inhibits (gates) gamma oscillations in task-irrelevant cortical areas. Indeed, synchronous gamma activity from higher-order (cognitive) to lower-order (sensory) cortical areas has been suggested as a possible mechanism of top-down attention control, whereby the processing of “meaningful” stimuli is facilitated (Baluch and Itti, 2011). The reduced alpha power that we observed for English compared to Jabberwocky around the same time and in similar posterior/vertex regions as the enhanced gamma band long-range phase synchrony may fit with this hypothesis. Future studies using cross-frequency coupling of alpha power with gamma phase synchrony could address this possible relationship further.

In contrast to our findings in the gamma and alpha frequency ranges, we did not find differences between English and Jabberwocky in the theta frequency range. Initially we were surprised by the absence of a between-condition effect, as a number of previous studies have reported increased theta band power when participants read sentence-embedded semantic violations compared to semantically unambiguous, correct words (Hagoort et al., 2004; Hald et al., 2006; Davidson and Indefrey, 2007; Willems et al., 2008; Wang et al., 2012; Bastiaansen and Hagoort, 2015). Increased theta band power has thus been linked to difficulty with lexical-semantic retrieval and integration (although see also Bastiaansen et al., 2002; Roehm et al., 2004; Pérez et al., 2012 for reports of increased theta power for sentence-embedded morphosyntactic violations relative to correct sentences as well). By this account, one might have predicted greater theta band power and/or phase synchrony for Jabberwocky compared to English, if the brain interpreted Jabberwocky as a series of lexical-semantic violations (pseudo-words). Instead, as part of our *post hoc* analyses, we found similar and significant increases in theta for both Jabberwocky and English, and no difference between conditions.

One possible explanation for these findings is that participants engaged in semantic processing for both sentence types, via the animated films that accompanied the sentences. These animations may have been enough for participants to follow the storyline and derive meaning from what they saw for both English and Jabberwocky, resulting in similar modulations to their theta band activity. However, if it were the case that the animations facilitated lexical-semantic retrieval and integration, we should have seen a decrease or no change in theta activity relative to baseline in our *post hoc* analyses, rather than an increase in theta for both conditions. Such a prediction would have followed from the results of previous studies involving sentence-embedded semantic violations (Hagoort et al., 2004; Hald et al., 2006; Davidson and Indefrey, 2007; Willems et al., 2008; Wang et al., 2012; Bastiaansen and Hagoort, 2015). Perhaps there is a fundamental difference in how brain activity changes to support semantic processing while reading semantic violation in otherwise-normal sentences, compared to our orally presented sentences that have no obvious semantic content, but which are presented alongside meaningful visual animations.

Alternatively, theta may reflect a more domain general mechanism to communicate a variety of information, rather than being specific to lexical-semantic processing *per se.* In fact, our finding of increased theta activity for both sentence types, followed by greater gamma activity for Jabberwocky alone both fits with and adds support to a number of recent proposals about the role of neural oscillations in speech perception more generally (Ghitza, 2011; Giraud and Poeppel, 2012; Peelle and Davis, 2012; Meyer, 2017). According to these theories, as the incoming speech signal becomes encoded in the auditory cortex, the phase of ongoing oscillations in the theta band resets to become aligned with the amplitude envelope of speech, whose rhythm is that of syllables, also around 4–7 Hz. This realignment is thought to enhance speech perception because the quasi-rhythmic features of syllable units in the input now arrive at a time when populations of relevant neurons are at the most excitable periods in their cycle (Peelle and Davis, 2012). Support for this theory comes from the finding of greater theta band phase synchronization for normal speech compared to speech whose acoustic envelope had been degraded to the point that the speech was no longer intelligible (Luo and Poeppel, 2007). Moreover, the fact that this phase synchronization occurs in the theta band (and is not a broadband response) underscores how it is the rhythm of speech *per se* that drives this realignment, rather than a general phase reset of ongoing oscillatory activity due to any stimulus onset (Mormann et al., 2005). What has still been unclear, however, is just how the brain’s phase locking of theta band oscillations to speech input might allow it to uncover meaning, in addition to lower-level acoustic information (Peelle and Davis, 2012; Meyer, 2017). In other words, does the brain’s ability to phase lock to acoustic cues in speech depend on the speech being intelligible?

Our *post hoc* findings of increased theta power and phase synchrony, relative to baseline for both English and Jabberwocky, raise the possibility of a more fundamental and general role for theta oscillations in speech perception. More specifically, theta’s acoustic envelope tracking may not depend directly on speech intelligibility, rather meaning may be derived indirectly from theta oscillations. Indeed slow-frequency theta oscillations are related to high-frequency gamma oscillations, which, due to their faster cycling rate, is thought to provide a more fine grained temporal integration window that is better suited for analysing sub-syllabic features in speech, such as phonemes and their combinations (Poeppel, 2003; Ghitza, 2011; Giraud and Poeppel, 2012). In our study, we found increased theta band power and phase synchrony relative to baseline for both meaningful English and meaningless Jabberwocky followed by greater gamma band power and phase synchrony for meaningful English only. The overall acoustic envelope of the English and Jabberwocky sentences was in fact similar, suggesting that intelligibility may not be a prerequisite for theta phase locking to occur. What differentiated English and Jabberwocky, was that Jabberwocky open-class content “words” were comprised of unfamiliar (although legal) combinations of phonemes that did not map onto a meaningful semantic representation. In other words, modulation of theta oscillations may reflect a domain-general tracking of the rhythm of the sentences’ syllabic structure that is similar regardless of whether the listener can uncover meaning from the speech signal. The brain may be predisposed to parse the approximately 4–7 Hz rhythm of speech (regardless of its semantic content), with oscillations tuned to the same frequency. Theta’s tracking of the acoustic envelope of speech may then provide a scaffold on which other temporal features of speech can be organized and, in the case of speech, meaning can be derived (Peelle and Davis, 2012). It appears that the brain differentiated meaningful English from meaningless Jabberwocky at the fine-grained phonetic level (and its mapping to semantic content), and this may be observable via modulations to its corresponding gamma rhythm. Theta rhythm, in contrast, may be how the brain communicates auditory information in general.

Together our findings suggest that long-range phase synchronization (functional connectivity), particularly in the gamma frequency band, may play an important role during meaningful speech perception. Phase synchronization has been proposed as a mechanism to explain the visual binding problem – how information in distributed brain regions can coordinate processing and communicate information across anatomically separate cortical areas in order to perceive a unitary visual percept (Varela et al., 2001). Analogously, during speech processing, the analysis of meaning requires not only spatial integration (as different types of linguistic information are processed by distributed brain regions) but also temporal integration as the speech signal unfolds over time (Hagoort, 2005). Dynamic functional connectivity, or brief periods of frequency-specific phase synchronization, as observed here, may provide a mechanism to help explain the language “binding” problem – how information retrieved from the mental lexicon over time can be unified with linguistic information processed by other brain areas into an overall coherent understanding of speech (Varela et al., 2001; Hagoort, 2005).

## Conclusion

In summary, our results suggest that the process of constructing a meaningful representation of incoming speech involves dynamic interactions among distributed brain regions that communicate through frequency-specific functional networks. In particular, phase synchronization of neuronal assemblies oscillating together at the gamma frequency range may provide a vehicle for information flow throughout a network of brain areas involved in extracting meaning in speech. Oscillations in the theta and alpha frequency ranges may also change during speech perception, although these changes may support more domain-general aspects of language, such as processing the acoustic or rhythmic features of speech (theta) and in gating activation of task-relevant brain areas (alpha). In contrast, our finding of greater long-range phase synchrony and local power in the gamma frequency range while participants listened to meaningful English compared to meaningless Jabberwocky speech, suggests that high-frequency gamma oscillations may reflect a mechanism by which the brain transfers and integrates semantic information in order for us to extract meaning and understand what is said.

## Ethics Statement

This study was carried out in accordance with the recommendations of the Research Ethics Board at the Hospital for Sick Children. The protocol was approved by the Research Ethics Board at the Hospital for Sick Children. All subjects gave written informed consent in accordance with the Declaration of Helsinki.

## Author Contributions

EW and EP designed the experiment. EW collected and analyzed all data. CN processed the data and assisted with analyses. AK programmed the matlab scripts to analyze the data. BD and TV verified the analytical methods and helped develop the theoretical framework. EW wrote the manuscript with input from EP and the other authors. EP supervised the project.

## Conflict of Interest Statement

The authors declare that the research was conducted in the absence of any commercial or financial relationships that could be construed as a potential conflict of interest. The reviewer MM declared a shared affiliation, with no collaboration, with several of the authors, BD and TV, to the handling Editor at time of review.
